# Rash-Negative Dermatomyositis-Spectrum Overlap Myositis With PM/Scl Positivity Presenting With Progressive Pharyngeal Dysphagia

**DOI:** 10.7759/cureus.109799

**Published:** 2026-05-28

**Authors:** Kevin Rivera, Caitlin Kesari

**Affiliations:** 1 Internal Medicine, Mount Carmel Health System, Columbus, USA; 2 Rheumatology, Mount Carmel Health System, Columbus, USA

**Keywords:** dermatomyositis, dysphagia, inflammatory myopathy, intravenous immunoglobulin, pm/scl antibody, systemic sclerosis overlap

## Abstract

Dermatomyositis-spectrum inflammatory myopathy may present without characteristic cutaneous findings, and overlap phenotypes can delay recognition when early neuromuscular examination is not yet diagnostic. We describe the case of a patient with rheumatology-diagnosed dermatomyositis without rash and PM/Scl-positive overlap myositis with systemic sclerosis features whose course was marked by interstitial lung disease, progressive weakness, and objectively confirmed pharyngeal dysphagia.

A 64-year-old female with interstitial lung disease presented with several weeks of polyarthralgia, severe fatigue, and marked muscle enzyme elevation. Initial examination showed no rash, sclerodactyly, or proximal weakness. Laboratory evaluation demonstrated a creatine kinase level of 12,810 U/L, elevated transaminases, a high-titer antinuclear antibody, positive PM/Scl-100 and PM/Scl-75 antibodies, and a weakly positive Mi-2 antibody. She was diagnosed with dermatomyositis without rash and PM/Scl-positive overlap myositis with systemic sclerosis features and treated with prednisone, mycophenolate mofetil, hydroxychloroquine, and antimicrobial prophylaxis. Intravenous immunoglobulin (IVIG) was recommended but was not initially approved. Her course progressed to severe weakness and dysphagia, ultimately preventing oral medication administration. During hospitalization, a videofluoroscopic swallow evaluation demonstrated aspiration with thin liquids and pharyngeal phase dysphagia. She received IVIG, with subsequent improvement in swallowing and functional status.

This report highlights the importance of an overlap-informed approach to inflammatory myopathy when classic cutaneous features are absent. Progressive dysphagia should be treated as a marker of severe disease because it increases the risk of aspiration, contributes to malnutrition, and may prevent the administration of oral immunosuppressive therapy.

## Introduction

Dermatomyositis is an idiopathic inflammatory myopathy classically associated with proximal muscle weakness, muscle enzyme elevation, and characteristic cutaneous findings. Contemporary classification frameworks incorporate clinical, laboratory, electromyographic, histopathologic, and cutaneous features, but bedside diagnosis often requires judgment when the clinical phenotype evolves over time or when hallmark findings are absent at presentation [1]. In this report, “rash-negative” dermatomyositis-spectrum disease refers to an inflammatory myopathy phenotype in which characteristic cutaneous findings are absent despite other supportive myopathic, serologic, and extramuscular features.

Dermatomyositis sine dermatitis is rare and is described largely through limited cohorts and case-based literature rather than through stable population-level prevalence estimates [2]. Although rash is central to the usual recognition of dermatomyositis, clinically meaningful disease may occur without classic cutaneous manifestations, creating diagnostic uncertainty when the initial presentation is dominated by constitutional symptoms, arthralgia, abnormal muscle-associated enzyme levels, or extramuscular involvement [2].

Diagnostic complexity increases further when inflammatory myopathy occurs as part of an overlap connective tissue disease. PM/Scl antibodies are associated with overlap myositis and may occur with systemic sclerosis features, often with prominent extramuscular disease [3,4]. In such patients, the presentation may not conform neatly to dermatomyositis, polymyositis, or systemic sclerosis in isolation. Interstitial lung disease is a major extramuscular manifestation of polymyositis and dermatomyositis, with a pooled global prevalence of approximately 41% in one meta-analysis, and may contribute substantially to morbidity and mortality [5]. For clinicians, this means that pulmonary disease, serologic findings, and muscle involvement must be interpreted together rather than as isolated abnormalities.

Dysphagia is another high-risk manifestation of inflammatory myopathy. It may lead to aspiration, malnutrition, impaired medication delivery, and functional decline [6]. In active dermatomyositis, systemic glucocorticoids and steroid-sparing agents remain central to treatment, with mycophenolate mofetil, calcineurin inhibitors, intravenous immunoglobulin (IVIG), rituximab, and other agents used according to phenotype, severity, and treatment response [7]. Randomized trial evidence supports the efficacy of IVIG in active dermatomyositis [8]. When progressive dysphagia prevents oral medication administration, escalation to parenteral therapy becomes not only a disease-control decision but also a practical necessity.

We describe a patient diagnosed by rheumatology with dermatomyositis without rash and PM/Scl-positive overlap myositis with systemic sclerosis features in the setting of PM/Scl-100 and PM/Scl-75 antibody positivity, weak Mi-2 antibody positivity, interstitial lung disease, marked prior creatine kinase elevation, and progressive pharyngeal dysphagia. Retrospective application of the 2017 European League Against Rheumatism/American College of Rheumatology criteria supported probable idiopathic inflammatory myopathy once later proximal weakness, enzyme elevation, and dysphagia were considered, although the absence of characteristic cutaneous findings limited formal classification as dermatomyositis by criteria alone [1]. The case illustrates how diagnostic confidence in overlap inflammatory myopathy may accumulate longitudinally and why dysphagia should prompt urgent reassessment of disease severity and treatment strategy.

## Case presentation

A 64-year-old female with interstitial lung disease and a history of toxic multinodular goiter, status post thyroidectomy and parathyroidectomy, presented with several weeks of polyarthralgia and severe fatigue. The pain involved the bilateral hands, elbows, shoulders, neck, and hips and was accompanied by diffuse upper extremity discomfort and generalized numbness. She described her legs as feeling “weighted,” with increasing difficulty ambulating, and reported bilateral hand swelling. She denied congestion, rhinorrhea, cough, wheezing, or cognitive symptoms.

On initial evaluation, she was afebrile with a blood pressure of 138/84 mmHg, a heart rate of 103 beats per minute, a respiratory rate of 14 breaths per minute, and a BMI of 26.5 kg/m². She appeared well nourished and in no acute distress. There was no alopecia, cutaneous rash, sclerodactyly, calcinosis, synovitis, psoriasis, nailfold capillary change, or obvious muscle atrophy. She had full fist formation bilaterally. The spine and paraspinal muscles were nontender, with normal curvature and range of motion. There was no sacroiliac joint tenderness or evidence of joint hypermobility. Gait was initially normal. Formal Manual Muscle Testing-8 was not performed at the first visit, but the rheumatologist documented proximal muscle strength to be full at 5/5 in the upper and lower extremities.

Initial laboratory evaluation during the early outpatient course demonstrated markedly elevated muscle enzymes, elevated aminotransferases, elevated inflammatory markers, and positive autoimmune serologies consistent with overlap inflammatory myopathy. Laboratory findings across the patient’s disease course are summarized in Table 1.

**Table 1:** Laboratory findings across the patient’s disease course Quantitative laboratory and serologic data are shown at the initial outpatient evaluation, approximately three months before admission, and at hospital admission. Elevated AST and ALT were interpreted in the context of marked CK elevation and normal total bilirubin, making muscle inflammation a plausible contributor rather than primary cholestatic hepatotoxicity ANA: antinuclear antibody; PM/Scl: polymyositis/scleroderma autoantibody; Mi-2: myositis-specific autoantibody associated with dermatomyositis; anti-Jo-1, PL-7, PL-12, EJ, and OJ: antisynthetase antibodies directed against aminoacyl-tRNA synthetases; SRP: signal recognition particle; TIF1-gamma: transcription intermediary factor 1-gamma; anti-MDA5: anti-melanoma differentiation-associated gene 5; NXP-2: nuclear matrix protein 2; U2 snRNP: U2 small nuclear ribonucleoprotein; anti-U1 RNP: anti-U1 ribonucleoprotein; SSA: Sjögren syndrome-related antigen A Table credits: Kevin Rivera, Caitlin Kesari

Laboratory parameter	Reference range	Initial outpatient evaluation, approximately three months before admission	Hospital admission	Interpretation
White blood cell count, ×10³/µL	4.6-10.2	6.4	3.5	Decreased
Hemoglobin, g/dL	12-16	13.5	11.1	Mildly decreased
Platelets, K/mcL	142-424	186	171	Normal
Sodium, mmol/L	136-145	136	135	Mildly decreased
Potassium, mmol/L	3.6-5.1	4.5	2.8	Decreased
Chloride, mmol/L	98-107	92	93	Decreased
CO_2_, mmol/L	22-32	29	31	Normal
Glucose, mg/dL	70-99	122	78	Elevated
Magnesium, mg/dL	1.8-2.5	1.2	1.4	Decreased
BUN, mg/dL	8-20	12	14	Normal
Creatinine, mg/dL	0.6-1.3	0.85	1.18	Normal
Creatine kinase (CK), U/L	34-145	12,810	524	Markedly elevated initially; improved but remained elevated at admission
Aspartate aminotransferase (AST), U/L	15-41	551	73	Elevated during the early outpatient course
Alanine aminotransferase (ALT), U/L	7-52	469	39	Elevated during the early outpatient course
Alkaline phosphatase, U/L	32-91	34	28	Decreased during admission
Albumin, g/dL	3.5-4.8	3.4	3.7	Decreased during outpatient
Total bilirubin, mg/dL	0.3-1.2	0.7	0.7	Normal
Erythrocyte sedimentation rate (ESR), mm/hr	0-30	55	Not repeated	Elevated
C-reactive protein (CRP), mg/L	0-1	23	Not repeated	Elevated
Antinuclear antibody (ANA)	Negative	1:1280, speckled	Not repeated	Positive
PM/Scl-100 antibody	Negative	Positive	Not repeated	Positive
PM/Scl-75 antibody	Negative	Positive	Not repeated	Positive
Mi-2 antibody	Negative	Weakly positive	Not repeated	Positive
Anti-Jo-1, PL-7, PL-12, EJ, OJ, SRP, TIF1-gamma, anti-MDA5, NXP-2, U2 snRNP, anti-U1 RNP, Ku, SSA, fibrillarin	Negative	Negative	Not repeated	Negative

Her pulmonary history was notable for persistent lung opacities followed by pulmonology. A chest CT obtained before hospitalization reportedly showed bilateral lower lobe consolidation and right middle lobe and lingular atelectasis versus consolidation, initially attributed to pneumonia. The abnormalities persisted despite antibiotic therapy. Tuberculosis and fungal cultures were negative. In the setting of PM/Scl antibody positivity and persistent pulmonary abnormalities, pulmonology treated her with high-dose corticosteroids for suspected connective tissue disease-associated interstitial lung disease. Echocardiographic evaluation by cardiology showed no evidence of pulmonary hypertension.

Rheumatology diagnosed dermatomyositis without rash and PM/Scl-positive overlap myositis with systemic sclerosis features. She did not meet the classification criteria for systemic sclerosis based on the available clinical findings. Still, PM/Scl antibody positivity, interstitial lung disease, and subsequent dysphagia supported an overlap phenotype. Three months before hospitalization, she was receiving prednisone 40 mg daily, mycophenolate mofetil 500 mg twice daily, hydroxychloroquine 400 mg daily, and trimethoprim-sulfamethoxazole prophylaxis three times weekly. IVIG was recommended early in the disease course, but insurance initially denied coverage.

Over the following weeks, she developed worsening fatigue and weakness severe enough to interfere with her activities of daily living, including driving. She required a cane for ambulation and had increasing difficulty rising from a chair and raising her arms overhead. Bilateral lower extremity edema also developed. One month before hospitalization, mycophenolate mofetil was changed to a liquid formulation because of worsening dysphagia, but she gradually lost the ability to tolerate oral medications.

She was admitted two months after the initial outpatient evaluation for progressive dysphagia, inability to take oral medications, worsening weakness, impaired dexterity, and severe protein-calorie malnutrition. On admission, vital signs included a temperature of 36.5 °C, heart rate of 98 beats per minute, respiratory rate of 14 breaths per minute, and blood pressure of 118/78 mmHg. She was alert and oriented but had generalized weakness. Laboratory testing demonstrated leukopenia, mild anemia, hypokalemia, hypomagnesemia, and improved yet persistently elevated creatine kinase (CK), as summarized in Table 1. Duplex ultrasound of the lower extremities showed no evidence of deep venous thrombosis.

**Figure 1:**
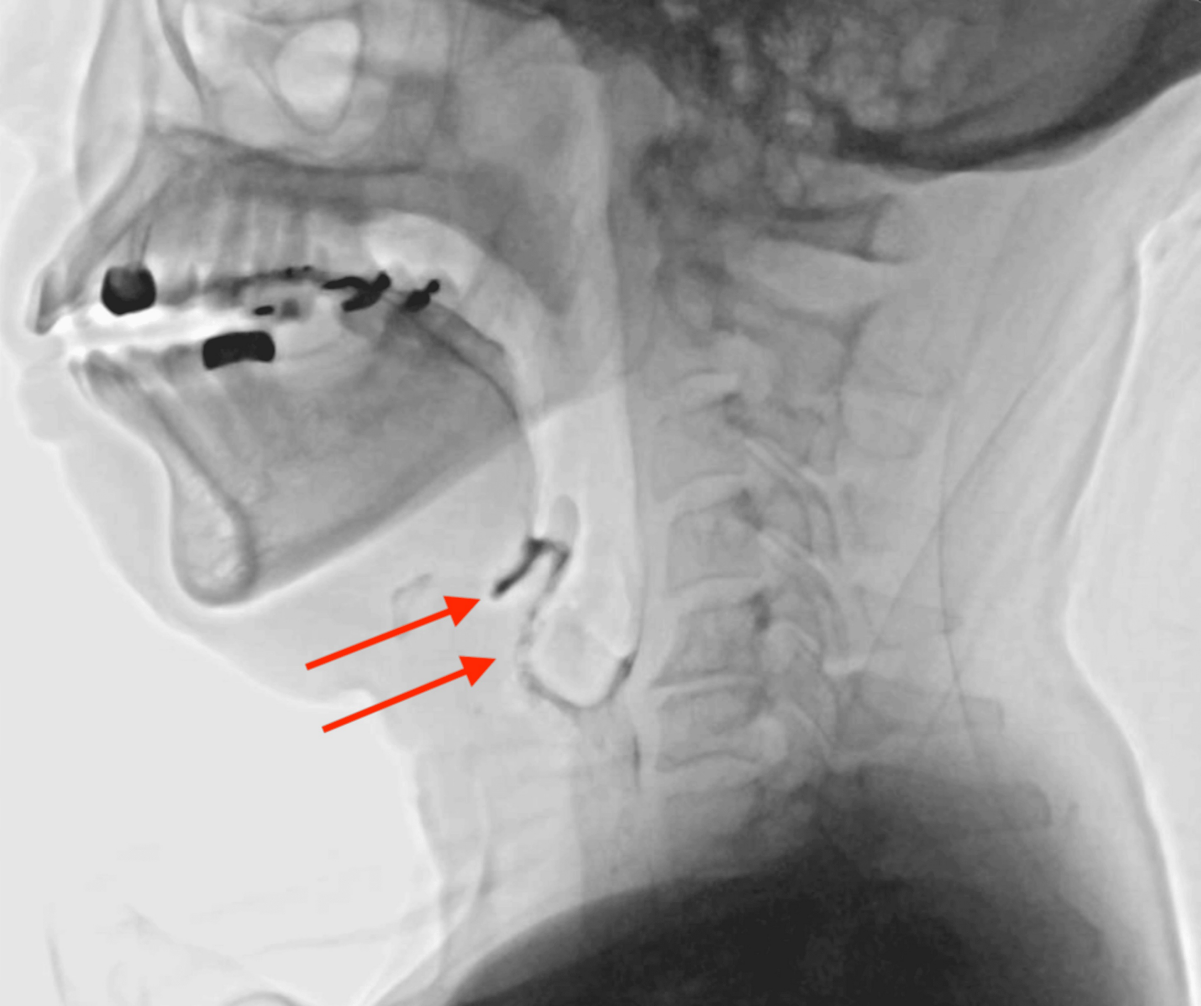
Modified barium swallow demonstrating pharyngeal dysphagia Representative still image from the video-fluoroscopic swallow study demonstrating penetration with one instance of sensate aspiration, premature spillage to the pyriforms, and vallecular/piriform residue. Arrows highlight sensate aspiration as well as vallecular and piriform residue Figure credits: Kevin Rivera, Caitlin Kesari

Dysphagia was evaluated with a modified barium swallow using video-fluoroscopic assessment in conjunction with speech-language pathology. The study demonstrated penetration with sensate aspiration, premature spillage into the pyriform sinuses, and mild-to-moderate vallecular residue, along with mild piriform residue. Speech-language pathology characterized this as mild-to-moderate pharyngeal phase dysphagia. A representative still image from the swallow study is shown in Figure 1.

**Figure 2:**
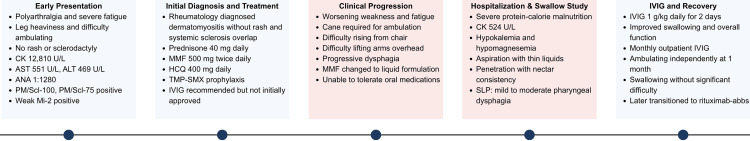
Clinical timeline of PM/Scl-positive dermatomyositis and systemic sclerosis overlap with progressive pharyngeal dysphagia The timeline shows the patient’s progression from early systemic and myopathic features to worsening dysphagia, inability to tolerate oral medications, hospitalization with objective swallow-study abnormalities, IVIG initiation, and subsequent functional recovery ANA: antinuclear antibody; CK: creatine kinase; HCQ: hydroxychloroquine; IVIG: intravenous immunoglobulin; MMF: mycophenolate mofetil; SLP: speech-language pathology; TMP-SMX: trimethoprim-sulfamethoxazole Figure credits: Kevin Rivera, Caitlin Kesari

She received IVIG at 1 g/kg once daily for two consecutive days and experienced clinical improvement. She was discharged with plans for continued therapy. At outpatient follow-up two weeks later, she reported improvement in weakness and fatigue. After insurance approval for ongoing therapy, she continued monthly IVIG and demonstrated continued functional recovery. At one month, she was ambulating independently and swallowing without significant difficulty. She ultimately received IVIG for one year and was later transitioned to rituximab-abbs 1000 mg every six months. The patient’s diagnostic and therapeutic course is outlined in Figure 2.

## Discussion

This case illustrates a diagnostic problem familiar to clinicians who care for inflammatory myopathy: early disease may be clinically active before the classic examination pattern is fully evident. The patient had marked CK elevation, elevated AST and ALT, systemic inflammation, high-titer ANA, PM/Scl-100 and PM/Scl-75 positivity, weak Mi-2 positivity, and pulmonary disease suggestive of connective tissue disease-associated interstitial lung disease. Yet the initial bedside strength examination was reassuring, and she had no cutaneous rash, sclerodactyly, calcinosis, nailfold capillary changes, or synovitis. Her CK decreased by approximately 96% between the initial outpatient evaluation and hospitalization after initiation of corticosteroid therapy, but she nevertheless developed worsening functional weakness and progressive pharyngeal dysphagia. This discordance underscores why improvement in muscle enzymes should not be interpreted as complete clinical control when bulbar symptoms and functional decline are still progressing.

The label “dermatomyositis without rash” should be used carefully because dermatomyositis is traditionally anchored by characteristic cutaneous manifestations. Rash-negative dermatomyositis-spectrum presentations have been described, but classification criteria do not replace longitudinal clinical reasoning in patients with evolving disease [1,2]. Using available data and no muscle biopsy, retrospective application of the 2017 European League Against Rheumatism/American College of Rheumatology criteria supported a diagnosis of probable idiopathic inflammatory myopathy once later proximal weakness, elevated muscle enzymes, and dysphagia were included. However, because heliotrope rash, Gottron papules, and Gottron sign were absent, the case could not be formally subclassified as dermatomyositis by criteria alone [1].

In this case, the working diagnosis was made by rheumatology and supported by several converging features: severe prior CK elevation, progressive functional weakness, weak Mi-2 positivity, PM/Scl antibody positivity, suspected connective tissue disease-associated interstitial lung disease, and objective pharyngeal dysphagia. These case-anchored findings are summarized in Table 2. The absence of muscle biopsy and electromyography limits phenotypic certainty, but it does not eliminate the practical diagnosis of inflammatory myopathy when the clinical course and serology are concordant and mutually supportive.

**Table 2:** Case-anchored findings supporting overlap inflammatory myopathy Clinical, serologic, pulmonary, bulbar, and treatment-response features supporting dermatomyositis-spectrum disease with systemic sclerosis overlap despite absent classic cutaneous findings CK: creatine kinase; AST: aspartate aminotransferase; ALT: alanine aminotransferase; IVIG: intravenous immunoglobulin Table credits: Kevin Rivera, Caitlin Kesari

Domain	Case finding	Clinical significance
Cutaneous findings	No rash, alopecia, sclerodactyly, calcinosis, or nailfold capillary change	Absence of classic cutaneous findings may delay recognition, but does not exclude inflammatory myopathy [1,2]
Muscle injury	CK 12,810 U/L early in the course; CK 524 U/L during hospitalization	Marked CK elevation strongly supported inflammatory myopathy, while the lower later value likely reflected partially treated disease [1,7]
Liver-associated enzymes	Elevated AST and ALT with normal total bilirubin	Muscle injury was a plausible contributor to aminotransferase elevation in the setting of marked CK elevation [7]
Serology	ANA 1:1280, PM/Scl-100 positive, PM/Scl-75 positive, weak Mi-2 positive	Supported PM/Scl-positive overlap myositis with dermatomyositis-spectrum and systemic sclerosis features [3,4]
Pulmonary context	Persistent lung opacities followed by pulmonology, without improvement after antibiotics	Supported concern for connective tissue disease-associated interstitial lung disease [5]
Bulbar involvement	Penetration with one instance of sensate aspiration and pharyngeal dysphagia on swallow evaluation	Penetration with one instance of sensate aspiration, premature spillage to the pyriforms, and pharyngeal residue on swallow evaluation
Treatment course	Improvement after IVIG initiation and continuation of outpatient therapy	Supported escalation to parenteral therapy when oral immunosuppression became impractical; IVIG is supported in active dermatomyositis [7,8]

The PM/Scl antibody profile was central to the interpretation of the overlap phenotype. PM/Scl antibodies are associated with overlap myositis and may occur with systemic sclerosis features, including pulmonary and esophageal involvement [3,4]. This patient did not have classic sclerodactyly, calcinosis, nailfold capillary abnormalities, telangiectasias, or pulmonary hypertension and therefore did not meet systemic sclerosis classification criteria based on available clinical findings. For that reason, we frame the case as PM/Scl-positive overlap myositis with systemic sclerosis features rather than definite systemic sclerosis. This distinction is important because overstating classic systemic sclerosis would misrepresent the case, while underrecognizing the PM/Scl overlap phenotype would miss the relevance of interstitial lung disease and swallowing involvement.

This distinction also helps separate the case from isolated polymyositis. Although the absence of rash could raise consideration of polymyositis, the weak Mi-2 positivity, PM/Scl-100 and PM/Scl-75 positivity, interstitial lung disease, and pharyngeal dysphagia supported a broader overlap inflammatory myopathy rather than pure polymyositis. In current practice, polymyositis is best approached as a diagnosis of exclusion after considering antibody-defined and overlap myositis phenotypes. The patient’s course, therefore, fits best as PM/Scl-positive overlap myositis with dermatomyositis-spectrum features rather than classic polymyositis or definite systemic sclerosis.

Pulmonary involvement also shaped the case. Interstitial lung disease is a major extramuscular manifestation of polymyositis and dermatomyositis, and its prevalence and outcome vary by antibody phenotype and population [5]. In this patient, pulmonary disease had been recognized before hospitalization and was followed by pulmonology after persistent lung opacities failed to improve with antibiotics, and infectious testing was unrevealing. Because the original outside chest imaging was not available for direct review, the radiographic pattern cannot be independently characterized here. Nevertheless, the pulmonary history remains clinically relevant because it contributed to the overlap diagnosis and influenced the treatment approach.

Dysphagia was the major inflection point. In inflammatory myopathy, dysphagia is not simply a symptom of discomfort. It can signal severe disease, increase aspiration risk, produce malnutrition, and prevent reliable administration of oral therapy [6]. This patient’s modified barium swallow demonstrated penetration with one instance of sensate aspiration, premature spillage into the pyriform sinuses, and vallecular and piriform residue. These findings localized the swallowing impairment to the pharyngeal phase and supported bulbar involvement from inflammatory myopathy. This is clinically distinct from isolated systemic sclerosis-associated esophageal dysmotility, which more typically reflects esophageal motility dysfunction rather than aspiration-producing pharyngeal phase impairment. Esophageal involvement could not be fully excluded without additional esophageal motility testing, but the objective swallow findings explained why oral medication administration became untenable. The functional consequences were clinically significant: inability to tolerate oral immunosuppression, severe protein-calorie malnutrition, hypokalemia, hypomagnesemia, and eventual hospitalization.

The therapeutic lesson is not that IVIG alone caused recovery, which cannot be proven from a single case. Rather, the case shows why stepwise oral immunosuppression may become insufficient once bulbar dysfunction progresses. Oral prednisone and mycophenolate mofetil require reliable swallowing, gastrointestinal delivery, and time to exert a therapeutic effect. Progressive pharyngeal dysphagia can therefore create both a disease severity problem and a drug delivery problem. Systemic glucocorticoids and steroid-sparing agents remain central to dermatomyositis treatment, with mycophenolate mofetil, calcineurin inhibitors, IVIG, rituximab, and other agents used according to phenotype, severity, and treatment response [7].

In the ProDERM trial, IVIG was associated with a significantly higher response rate than placebo in adults with active dermatomyositis, supporting its role as an evidence-based treatment option in active disease [8]. In this patient, IVIG was recommended early but delayed due to a lack of insurance approval. During that interval, weakness and dysphagia progressed. After IVIG was administered, she improved clinically, later continued monthly therapy, and recovered the ability to ambulate independently and swallow without significant difficulty. In this context, IVIG was not only a treatment for active inflammatory myopathy but also a practical parenteral strategy when oral therapy had become unreliable. Practical management considerations raised by this clinical course are summarized in Table 3.

**Table 3:** Practical management considerations when dysphagia develops in inflammatory myopathy Clinical implications of progressive dysphagia, including reassessment of disease severity, objective swallow evaluation, supportive care, and escalation when oral therapy becomes unreliable Table credits: Kevin Rivera, Caitlin Kesari

Clinical problem	Management implication	Rationale
Progressive weakness despite glucocorticoids	Add or optimize steroid-sparing immunosuppression	Functional decline suggests inadequate disease control and a need for treatment escalation [7]
PM/Scl-positive overlap phenotype	Monitor for pulmonary, swallowing, and esophageal involvement	PM/Scl-associated overlap disease may be characterized by prominent extramuscular manifestations [3,4]
Progressive dysphagia	Obtain an objective swallow evaluation and reassess disease severity	Dysphagia increases aspiration risk, contributes to malnutrition, and may indicate more severe myositis involvement [6]
Inability to tolerate oral medications	Expedite parenteral therapy	Oral therapy may fail when it cannot be administered or absorbed reliably [7]
Aspiration or penetration on swallow study	Coordinate speech therapy, nutrition support, and immunomodulatory treatment	Dysphagia management requires both supportive and disease-directed care [6,7]
Improvement after escalation	Continue effective maintenance therapy with close follow-up	Sustained disease control is needed to preserve functional recovery and reduce relapse risk [7,8]

Several limitations of this report should be acknowledged. Muscle biopsy and electromyography were not pursued, limiting histopathologic confirmation and the ability to precisely classify the inflammatory myopathy subtype. Formal Manual Muscle Testing-8 was not performed at the first visit, limiting the reproducibility of the initial strength assessment. The patient did not meet systemic sclerosis classification criteria based on available clinical findings, so the overlap designation rests on PM/Scl antibody positivity, pulmonary involvement, swallowing involvement, and rheumatology assessment rather than definite systemic sclerosis classification. The outside chest imaging that supported the pulmonary component of the overlap phenotype was not available for direct review, and pulmonary function testing during the period of clinical decline was not available, limiting full characterization of interstitial lung disease activity. Although the patient's condition worsened during a period when IVIG was not accessible and improved after IVIG initiation, causality cannot be inferred from a single patient observation. Finally, because this was a real-world clinical course rather than a protocolized diagnostic evaluation, the diagnosis depended on longitudinal synthesis of clinical trajectory, serologic profile, exclusion of competing explanations, and therapeutic response.

Despite these limitations, the practical message is clear. Inflammatory myopathy should remain in the differential diagnosis when systemic inflammation, marked CK elevation, and unexplained aminotransferase elevation occur, even if rash is absent and early strength testing is preserved. PM/Scl antibody positivity should prompt attention to overlap features, especially pulmonary and swallowing involvement. Most importantly, progressive dysphagia should be treated as a marker of disease severity because it may transform a manageable outpatient immunosuppressive regimen into an urgent need for speech-language pathology evaluation, objective swallow testing, nutritional support, and parenteral therapy.

## Conclusions

Dermatomyositis-spectrum inflammatory myopathy and overlap myositis may present without classic cutaneous findings, and early examination may underestimate disease severity. In this patient, PM/Scl-positive overlap myositis with dermatomyositis-spectrum features was supported by marked prior CK elevation, PM/Scl-100 and PM/Scl-75 antibody positivity, weak Mi-2 positivity, suspected connective tissue disease-associated interstitial lung disease, progressive weakness, and objective pharyngeal dysphagia. Dysphagia was the key clinical turning point because it increased aspiration risk, contributed to malnutrition, and compromised reliable oral medication administration. In PM/Scl-positive overlap inflammatory myopathy, progressive dysphagia should prompt urgent speech-language pathology assessment, objective swallow evaluation, nutritional support, and consideration of intravenous induction therapy, such as IVIG or pulse corticosteroids, rather than waiting for oral medications to take effect alone.
